# The upper thermal limit of epaulette sharks (*Hemiscyllium ocellatum*) is conserved across three life history stages, sex and body size

**DOI:** 10.1093/conphys/coac074

**Published:** 2022-12-28

**Authors:** Carolyn R Wheeler, Bethan J Lang, John W Mandelman, Jodie L Rummer

**Affiliations:** ARC Centre of Excellence for Coral Reef Studies, James Cook University, Townsville, Queensland 4814, Australia; School for the Environment, The University of Massachusetts Boston, Boston, MA 02125, USA; ARC Centre of Excellence for Coral Reef Studies, James Cook University, Townsville, Queensland 4814, Australia; School for the Environment, The University of Massachusetts Boston, Boston, MA 02125, USA; Anderson Cabot Center for Ocean Life, New England Aquarium, Boston, MA 02110, USA; ARC Centre of Excellence for Coral Reef Studies, James Cook University, Townsville, Queensland 4814, Australia; College of Science and Engineering, James Cook University, Townsville, Queensland 4814, Australia

**Keywords:** thermal tolerance, ocean warming, epaulette shark, critical thermal maximum, Chondrichthyes

## Abstract

Owing to climate change, most notably the increasing frequency of marine heatwaves and long-term ocean warming, better elucidating the upper thermal limits of marine fishes is important for predicting the future of species and populations. The critical thermal maximum (CT_max_), or the highest temperature a species can tolerate, is a physiological metric that is used to establish upper thermal limits. Among marine organisms, this metric is commonly assessed in bony fishes but less so in other taxonomic groups, such as elasmobranchs (subclass of sharks, rays and skates), where only thermal acclimation effects on CT_max_ have been assessed. Herein, we tested whether three life history stages, sex and body size affected CT_max_ in a tropical elasmobranch, the epaulette shark (*Hemiscyllium ocellatum*), collected from the reef flats surrounding Heron Island, Australia. Overall, we found no difference in CT_max_ between life history stages, sexes or across a range of body sizes. Findings from this research suggest that the energetically costly processes (i.e. growth, maturation and reproduction) associated with the life history stages occupying these tropical reef flats do not change overall acute thermal tolerance. However, it is important to note that neither embryos developing *in ovo*, neonates, nor females actively encapsulating egg cases were observed in or collected from the reef flats. Overall, our findings provide the first evidence in an elasmobranch that upper thermal tolerance is not impacted by life history stage or size. This information will help to improve our understanding of how anthropogenic climate change may (or may not) disproportionally affect particular life stages and, as such, where additional conservation and management actions may be required.

## Introduction

Ectothermic fishes are dependent on environmental water temperature to regulate all aspects of their biology, where organisms have optimal temperature ranges for their growth, reproduction and ultimately their survival (Angilletta Jr, 2009). When temperatures shift outside of optimal ranges, negative consequences arise for individuals that can cascade to population level issues and overall ecosystem health (e.g. Angilletta Jr, 2009; Pörtner and Peck, 2010). With the increasing occurrence of marine heatwaves and sustained ocean warming due to anthropogenic climate change (Fox-Kemper *et al.,* 2021), the biological thermal constraints of marine ectothermic fishes are of growing concern (Pörtner and Peck, 2010). Therefore, it is imperative that we assess thermal ranges and end points (minima and maxima) to understand current thermal constraints and aid our understanding of the role that thermal plasticity may play in a warming ocean (Angilletta Jr, 2009; Gunderson and Stillman, 2015; Seebacher *et al.,* 2015). To do so, one physiological metric, critical thermal maximum (CT_max_), which marks an organism’s highest acute temperature tolerance, is commonly established by incrementally increasing water temperatures until the individuals lose motor function or the onset of muscle spasms occurs (Lutterschmidt and Hutchison, 1997; Kingsolver and Umbanhowar, 2018). Although the high temperatures reached during these tests for most species do not reflect non-captive ecological scenarios, they provide a measure of the upper thermal capability of a species that can be repeated and compared intra- and inter-specifically (Morgan *et al.,* 2018).

For elasmobranch fishes (sharks, rays and skates), aside from being one of the most globally threatened taxa due to fisheries interactions (Dulvy *et al.,* 2021), ocean warming has also become of concern (Pereira Santos *et al.,* 2021). This subclass of mostly ectothermic fishes exhibits slow generation times on the order of years to decades (Conrath and Musick, 2012), thus reducing their potential for thermal transgenerational adaptation (Pereira Santos *et al.,* 2021). Furthermore, many elasmobranch species are demersal and exhibit a degree of site fidelity (e.g. Awruch *et al.,* 2012; Kneebone *et al.,* 2020), potentially limiting their ability to undertake large-scale migrations to relocate to cooler, more suitable thermal conditions that also provide adequate benthic habitat (Vilmar and Di Santo, 2022). So, despite mounting global concerns for elasmobranch populations, and that their life history strategies may preclude their ability to quickly adapt to changing environments, the thermal biology of most elasmobranch species remains under-investigated (Pereira Santos *et al.,* 2021). For example, CT_max_ estimates from elasmobranchs are limited to only four species and have only been assessed in relation to thermal history (Fangue and Bennett, 2003; Dabruzzi *et al.,* 2013; Gervais *et al.,* 2018; Bouyoucos *et al.,* 2020).

Different life history stages, because of their associated energetic demands, may be differentially impacted by warming and temperature stress (Pörtner and Farrell, 2008; Przeslawski *et al.,* 2015; Dahlke *et al.,* 2020). During life stages where growth, development and gamete production result in high energetic costs, organisms are hypothesized to exhibit reduced upper thermal tolerance (Dahlke *et al.,* 2020). There may also be inherent thermal tolerance differences between sexes and across increasing mass over life stages, as large-bodied fishes heat internally more slowly, producing higher CT_max_ values (Stevens and Fry, 1974; Zhang and Kieffer, 2014; Messmer *et al.,* 2017). However, these hypotheses have never been tested in an elasmobranch species, where the time scale of development and reproduction can span months to years (Conrath and Musick, 2012), representing large energetic costs. Moreover, species from this taxon generally have larger body mass ranges compared to teleost species investigated in most CT_max_ studies (e.g. Ospina and Mora, 2004; Messmer *et al.,* 2017; Morgan *et al.,* 2018, 2019; Firth *et al.,* 2021; Penney *et al.,* 2021).

To understand the influence that life history stage, sex and body size have on the thermal tolerance of elasmobranchs, we studied the epaulette shark (*Hemiscyllium ocellatum*), which is a small demersal shark found throughout the Great Barrier Reef (GBR), Australia (Dudgeon *et al.,* 2019). This tropical species inhabits shallow, coastal environments that vary daily but reside within a narrow thermal niche, where they may experience (on average) only 6–7°C of water temperature change annually (Heupel *et al.,* 1999; Nay *et al.,* 2021). Furthermore, epaulette sharks are abundant in multiple life stages (juveniles to adults) on reef flats throughout the GBR, and the relationship between body size and maturity has been previously established (Heupel *et al.,* 1999). Epaulette sharks are oviparous (egg-laying), reproducing annually from July to December (Heupel *et al.,* 1999). Therefore, this species allows us to assess thermal limits within a singular season to mitigate seasonal acclimatization effects (Gervais *et al.,* 2018) while still using a range of life history stages, including reproducing adults.

We hypothesized that reproducing adults may have a reduced thermal tolerance when compared to other life stages, as high energetic investments toward gamete production likely reduce the amount of energy available for thermal stress responses (e.g. Freitas *et al.,* 2010; Komoroske *et al.,* 2014; Clark *et al.,* 2017; Dahlke *et al.,* 2020). Furthermore, we hypothesize that the upper thermal tolerance of juveniles and subadults would be similar between sexes, but mature adult females would have a lower thermal tolerance than mature adult males, given that female reproduction appears to be more costly than male reproduction (Hayward and Gillooly, 2011). Finally, we hypothesized that larger body mass individuals would have a higher upper thermal tolerance when compared to smaller conspecifics. Overall, outcomes from this study will inform whether certain life history stages in the epaulette shark—and potentially other similar species—are more vulnerable to warming, which is important information for current and future frameworks of elasmobranch conservation under the threat of ocean warming (Pereira Santos *et al.,* 2021).

## Methods

### Animal ethics and permits

The experimental protocols in this study were approved by the James Cook University Animal Ethics Committee (protocol A2739). The temporary collections were conducted under the appropriate Queensland Fisheries (#255136) and Great Barrier Reef Marine Park Authority (GBRMPA G21/44922.1) permits.

### Collections and temporary holding

Thirty epaulette sharks were collected from the Heron Island reef flat (23.4423°S, 151.9148°E) during low tides in October and November 2021 during the peak reproductive season (Heupel *et al.,* 1999). Sharks were hand-caught with dip nets, sexed and photographed for their unique spot pattern for identification purposes and transported back to the Heron Island Research Station. The individuals were temporarily maintained for two to seven days in a 364 cm by 364 cm aquarium (flow-through system) supplied with 4770 litres of seawater from the reef flat. The aquarium was located outside with natural photoperiod and diel changes in water temperature that mirrored the reef flat where the sharks were collected. Sharks were not fed during their time in captivity so that experimentation could commence with animals that had been fasted for at least 48 hours. No more than 15 sharks were in captivity at any given time, and sharks were provided with shelters, given their propensity to hide in their natural habitat.

### Critical thermal maximum assays

To determine CT_max_, a 156 cm diameter by 56 cm high cylindrical aquarium was filled with 363 litres of clean seawater at the same temperature as the holding aquarium, and a weighted basket made of plastic mesh measuring 60 cm by 42 cm by 42 cm was placed inside to restrict movement of the sharks. Thermal ramping rate, or how quickly the water is warmed during a CT_max_ assay, can impact the final endpoint, where slower thermal ramping rates produce lower CT_max_ values and vice versa (Messmer *et al.,* 2017; Kingsolver and Umbanhowar, 2018; Illing *et al.,* 2020). Therefore, it is important to choose a rate that allows the internal body temperatures to increase in parallel, which is especially important for larger fish species, given the body mass differential, but fast enough such that it precludes acclimation (Messmer *et al.,* 2017; Kingsolver and Umbanhowar, 2018; Illing *et al.,* 2020). We used a 2000-watt titanium heater to heat the water at a rate of 0.1°C min^−1^, which is slower than previous CT_max_ elasmobranch studies (i.e. 0.3°C min^−1^: Fangue and Bennett, 2003; 0.25°C min^−1^: Dabruzzi *et al.,* 2013; 0.26°C min^−1^: Gervais *et al.,* 2018; 0.28°C min^−1^: Bouyoucos *et al.,* 2020), but likely an improved rate for this generally large-bodied taxon. Both water temperature and oxygen concentrations were logged every five seconds throughout the assays with an OXROB3 fibre optic probe connected to a Firesting Optical Oxygen Meter (Pyroscience GmbH, Aachen, Germany). An air stone and two 1000 litre hr^−1^ pumps were used to ensure homogeneous mixing of water throughout the experimental aquarium during assays, and oxygen concentrations did not drop below 80% air saturation. Starting water temperature of the assays ranged from 22.8 to 26.7°C, but could not be controlled for, as inflowing water came directly from the reef flat, which warms throughout the day. As a result, morning assays started at lower temperatures than afternoon assays.

Sharks were allowed to habituate to the experimental aquarium for one hour prior to thermal ramping (Gervais *et al.,* 2018), and we ensured the sharks were at rest for at least 10 minutes prior to the start of the assay. Ventilation rates (gill beats per min; bpm) were measured every 15 minutes for the first part of the assays, while rates were increasing; then, after ventilation rates peaked and began to remain constant or decrease, measurement frequency increased to every two minutes (Gervais *et al.,* 2018). At each ventilation count, it was noted whether the shark was active or resting. After each of the two-minute interval ventilation measurements, the sharks were flipped ventrally 180 degrees along the longitudinal axis to test for the loss of righting reflex (LRR; Lutterschmidt and Hutchison, 1997; Gervais *et al.,* 2018). Sharks were considered to have lost their righting reflex when they were unable to right themselves within ten seconds (Zhang and Kieffer, 2014). After LRR was reached, sharks were immediately removed to a water bath at the initial starting temperature of the assay, and the end point temperature of LRR was recorded. Sharks were allowed to recover for 24 hours after the assays were conducted.

### Life history staging

**Figure 1:**
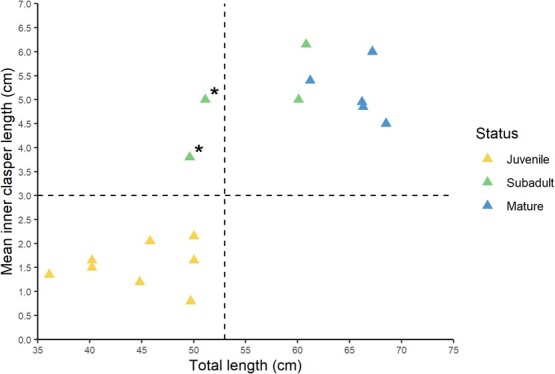
Male epaulette shark total length (cm) verses average inner clasper length (cm) assessed in this study. The horizontal and vertical dashed lines respectively represent the cut-off between juvenile and subadult clasper length and total length. Two sharks (denoted with *) had total lengths indicative of juvenile status but also had elongated and partially calcified claspers and were therefore deemed subadults

After recovery, sharks were measured for total length (TL; cm), mass (kg) and, for males, inner clasper length. Female sharks were palpated to detect the presence or absence of egg cases. Sharks were classified into three life history stages of juvenile, subadult or mature adult, based on total lengths reported in Heupel et al. (1999; Table 1). Similar to findings from Heupel et al. (1999), males in the 49–61 cm TL range had claspers that ranged in length and calcification status (Fig. 1), and so we prioritized clasper length and calcification over TL when categorizing male life history stages. For example, males are considered juveniles when TL is less than 53 cm, but two of the small males in this study (49.6 and 51.1 cm TL) also had partially elongated and calcified claspers that were 3.8 and 5 cm, respectively (asterisk marked data points, Fig. 1). Therefore, we classified these sharks as subadults and not juveniles, as maturation had clearly begun.

**Table 1:** Life history stage classification of epaulette sharks (*H. ocellatum)* from sex-specific total lengths and clasper lengths at maturity, as reported in Heupel et al. (1999)

Life history stage	Total length (cm)
Juvenile	M: <53 cm
	F: <55 cm
Subadult	M: 53–61 cm TL **OR** inner clasper length ≥3.0 cm
	F: 55–61 cm
Mature adult	>61 cm

Finally, each shark was implanted with a passive integrated transponder tag into the dorsal musculature to the left of the first dorsal fin to ensure no shark was re-captured and repeated in our experiments. All sharks were released onto the reef flat at the location of capture in good health (e.g. exhibiting normal ventilation rates and behaviour, and no change in overall coloration or redness on the ventral side, as per Gendron and Menzies, 2004). Of the 30 sharks assessed for CT_max_, nine were juveniles (8 M; 1 F), eight were subadults (4 M; 4 F) and 13 were mature adults (5 M; 8 F; Table 2). No females were encapsulating egg cases at the time of capture or at the time of CT_max_ assays.

**Table 2:** The mean CT_max_, standard deviation (s.d.) and range for each life history stage and sex with one outlier removed (see Table S0)

Life history stage	Mean mass (kg) (±s.d.) [range]	Mean total length (cm) (±s.d.) [range]	Mean CT_max_ (°C) (±s.d.)	n
Juvenile	0.26 ± 0.10 [0.11–0.39]	44.6 ± 5.0 [36.1–50.0]	36.14 ± 0.26	9
Subadult	0.47 ± 0.10 [0.35–0.62]	56.3 ± 4.1 [49.6–60.8]	36.17 ± 0.35	8
Mature adult	0.67 ± 0.07 [0.54–0.64]	65.6 ± 2.8 [61.2–66.3]	36.20 ± 0.27	12
All females	0.58 ± 0.18 [0.18–0.78]	61.0 ± 6.8 [45.0–70.3]	36.24 ± 0.23	12
All males	0.42 ± 0.19 [0.11–0.74]	53.4 ± 10.4 [36.1–68.5]	36.13 ± 0.30	17

### Statistical analyses

First, one outlier was identified by a Dixon’s Q test and was removed from the dataset (*Q* = 0.50595, *P* = 0.001172) (Supplementary S0) (Komsta, 2022). To test for the effects of non-controlled trial conditions, a linear regression was performed incorporating the number of days each shark was in holding at the field station prior to the CT_max_ trials. Additionally, the time of day of the start of the trial and the starting water temperature were included in an interaction term to account for changes in water temperature across the course of the day (Supplementary Table S1). Next, a linear model was fit to assess the effect of life history stage, sex and mass on CT_max_, where all factors were included in an interaction term to account for inherent relationships between these factors (e.g. mature sharks have higher mass) (Supplementary Table S2).

To assess how activity level during CT_max_ trials differed between life history stages and sex, we performed a binomial (i.e. active vs resting) generalized linear mixed-effects model (GLMM) with individual as a random effect (Supplementary Table S3) (*lme4*; Bates *et al.,* 2015). Next, to assess ventilation rates across CT_max_ trials, we considered activity level findings from the previously described GLMM model. Although not significant, some subadults were more active during trials, which artificially inflated the subadult overall ventilation rate model fits when data from both activity levels were included. Additionally, we were not able to quantify the duration or magnitude of swimming activity across the trial or at each observation, where fast or sustained swimming would increase ventilation rates more quickly over time. Therefore, we have only included ventilation rates from resting sharks herein, so data are directly comparable between groupings. We performed a generalized additive model (GAM) of ventilation rate over a smoother of the experiment time with an interaction term for life history stage and sex (*mgcv*; Wood, 2011) (Supplementary Table S4). We also included the first order auto-correlation function to account for the fact that ventilation rates are inherently dependent on the previously measured rate (Supplementary Table S4). Finally, the estimated marginal means within each sex were compared between life history stages (*emmeans*; Lenth, 2022) (Supplementary Table S4). All models were visually checked for normality and homoscedasticity using qqplots and residual plots, and all analyses were conducted in R (version 4.1.1., R Core Team, 2021).

## Results

**Figure 2:**
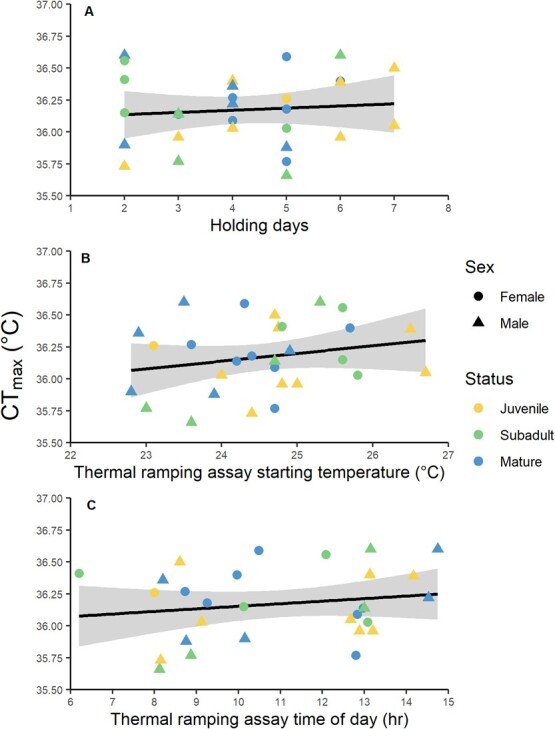
Uncontrolled experimental conditions that could impact CT_max_. There was no effect of (A) number of days in captivity prior to assays (*P* = 0.57), (B) starting water temperature of the assays (*P* = 0.48) or (C) time of day when assay commenced (*P* = 0.68) on CT_max_. Shaded areas represent the 95% confidence intervals of the linear models

**Figure 3:**
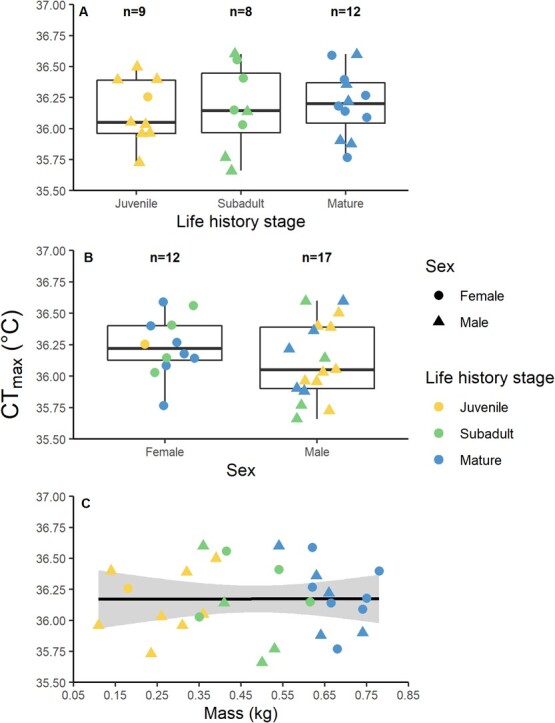
Boxplots and linear regression of the mean CT_max_ across (A) life history stages (*P* = 0.56), (B) sex (*P* = 0.11) and (C) body mass (*P* = 0.26). The shaded area represents the 95% confidence intervals of the linear fit

Despite the assay starting water temperature ranging from 22.8 to 26.7°C, this factor did not affect the CT_max_ end point (*F*_1,24_ = 0.50, *P* = 0.48; Fig. 2B). Furthermore, the number of holding days before the assay (*F*_1,24_ = 0.33, *P* = 0.57; Fig. 2A), the time of day of the assay (*F*_1,24_ = 0.18, *P* = 0.68; Fig. 2C), nor the interaction term (*F*_1,24_ = 1.42, *P* = 0.25) had any effect on CT_max_; these factors were therefore excluded from the subsequent CT_max_ models. CT_max_ did not change across the distinct life history stages of juveniles, subadults or mature adults (*F*_2,18_ = 0.60, *P* = 0.56; Table 2, Fig. 3A). Furthermore, CT_max_ did not differ between sexes (*F*_1,18_ = 2.80, *P* = 0.11; Table 2, Fig. 3B) or over body mass (*F*_1,18_ = 1.33, *P* = 0.26; Table 2, Fig. 3C). This model did include significant interactions between life history stage and mass (*F*_2,18_ = 4.56, *P* = 0.02) as well as sex and mass (*F*_1,18_ = 6.03, *P* = 0.02) (Supplementary Table S2).

**Figure 4:**
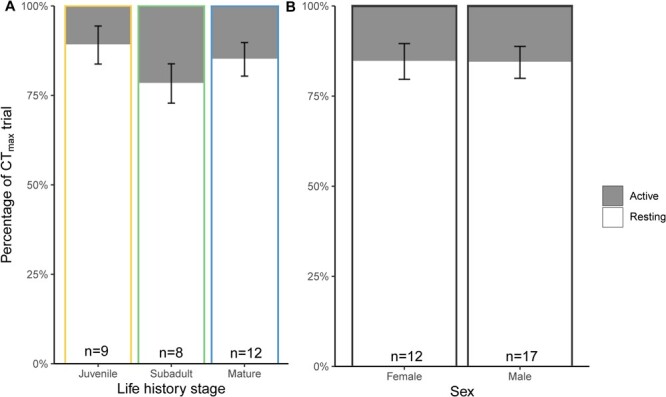
The proportion of activity type (resting verses active) during CT_max_ trials across (A) life history stages and (B) between sexes. Error bars represent standard error of the mean and there was no significant difference between any groupings (life history stage: *P = 0.21*; sex: *P = 0.45*)

**Figure 5:**
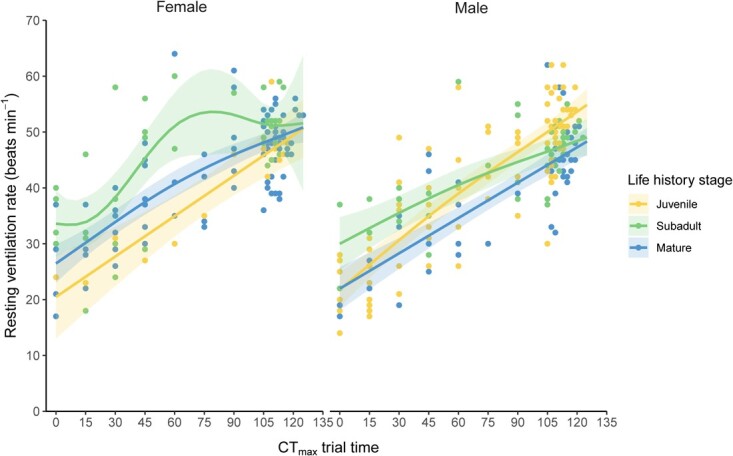
Ventilation rates (gill beats per minute) of epaulette sharks across CT_max_ assays between (A) life history stages and between (B) sexes. Ventilation rates were measured every 15 minutes for the first 105 minutes, and then every two minutes until the loss of righting reflex occurred. Shaded areas represent the 95% confidence intervals of the linear mixed effects model fits

During CT_max_ trials, there was no difference in the proportion of time spent resting verses active between life history stages (χ^2^ = 3.14, *P* = 0.21; Fig. 4A) or sexes (χ^2^ = 0.58, *P* = 0.45; Fig. 4B) (Supplementary Table S3). The resting ventilation rates of sharks did not differ between sexes (*F* = 3.87, *P* = 0.05, Fig. 5) (Supplementary Table S4). Within females, subadults had higher ventilation rates than juveniles (t-ratio = −3.44, *P* = 0.00) and adults (t-ratio = 2.42, *P* = 0.04), and within males, both juveniles (t-ratio = 3.81, *P* = 0.00) and subadults (t-ratio = 3.01, *P* = 0.01) differed from mature adults (Fig. 5, Supplementary Table S4).

## Discussion

With the aim to determine whether three life history stages, sex and body size affected the upper thermal tolerance limits of a tropical elasmobranch species, this study did not find any effects of these factors on CT_max_ (Table 2, Fig. 3). Although CT_max_ trials were not identical in terms of animal holding time (i.e. time from temporary collection to the CT_max_ trial), starting water temperature or time of day, we found no effect of these experimental design variables on CT_max_ (Fig. 2). Finally, we did find small differences in the resting ventilation rates during CT_max_ trials, particularly within males (Fig. 5B). Our findings fuel the question: how are various life history stages and each sex impacted by thermal stress if it is not reflected in their upper thermal tolerance? Moreover, what are the most useful physiological markers to inform this question in future research? Answering these questions will provide a more uniform approach amongst researchers working to understand thermal limits in the context of climate change.

### Life history stage and sex effects on CT_max_

Having synthesized thermal limits across teleost fishes, Dahlke et al. (2020) proposed that thermal tolerance differs across life history stages. During embryonic stages of development, individuals do not have a fully developed cardiorespiratory system, and embryos would have a reduced capacity to circulate oxygen to tissues (Pörtner and Peck, 2010). Furthermore, in spawning adults, energy is diverted to gamete development, thus reducing the amount of oxygen available for a thermal response (Pörtner and Peck, 2010; Dahlke *et al.,* 2020). These hypotheses are well reflected in the synthesized data; however, Pottier et al. (2022) has contested these findings, indicating that the thermal threshold data were compared between different methodologies (e.g. CT_max_ verses field temperatures), where some datasets were likely underestimating upper thermal tolerance in embryos and adults. Indeed, many studies including multiple life stages have not found differences in CT_max_ (e.g. Ospina and Mora, 2004; Recsetar *et al.,* 2012; Andreassen *et al.,* 2022); however, including a full range of life stages within a singular study is challenging, as most studies are lacking the very earliest life stages or validated reproducing adults (Johnson, 1976; Ospina and Mora, 2004; Recsetar *et al.,* 2012; Andreassen *et al.,* 2022).

In the context of the current study, we were not able to include embryonic and neonate life stages, as egg cases and newly hatched neonate sharks have not been documented on the Heron Island reef flat (Heupel *et al.,* 1999 and this study). Including these earlier life stages could help elucidate if a bottleneck in CT_max_ exists in this species. However, embryonic CT_max_ would likely require a different endpoint metric than LRR used here and across many other studies, given that elasmobranch embryos are attached to a large yolk and do not necessarily maintain a specific orientation and would therefore not right themselves. Perhaps cessation of other motor functions such as tail movements during early developmental stages and the onset of muscle spasms during later developmental stages would suffice. However, these endpoints are likely not directly comparable to LRR, as muscle spasms, for example, occurs well after LRR, which is why studies using that endpoint tend to produce higher CT_max_ estimates (Lutterschmidt and Hutchison, 1997).

In this study, we were also unable to assess CT_max_ in females that were actively encapsulating egg cases. The egg encapsulation process for this species is only 2–5 days long (Wheeler, unpublished data), which is difficult to capture when collecting wild sharks. However, adult sharks in this study were presumably undergoing other reproductive processes beyond egg encapsulation during our experiments, such as spermatogenesis for males and vitellogenesis and folliculogenesis for females. Indeed, we conducted this study during the reproductive season (Heupel *et al.,* 1999), and ejaculate was found on several calcified claspers of adult males during physical exams. Although nothing is known of reproductive effects on CT_max_ in elasmobranchs, there are some findings in viviparous teleosts, although limited and conflicting. Auer *et al.* (2021) reported that CT_max_ was similar from pre-gestation to early gestational stages but decreased by nearly 0.5°C in late stages prior to parturition. Contrarily, Johnson (1976) found no difference in CT_max_ between gravid and non-gravid female counterparts, but those females overall had higher CT_max_ estimates than males. More research comparing reproductive effects on CT_max_ through stages of both oviparity and viviparity as well as the effects of maternally experienced thermal stress on the health of offspring would be pertinent future research.

As water temperatures increase, ectotherms increase aerobic oxygen uptake rates to a point before switching to unsustainable anaerobic pathways (Pörtner and Peck, 2010). So, it follows that we observed increases in ventilation rates with increasing temperatures during experimentation (Angilletta Jr, 2009). Given that all life stages in this study had fully developed cardiorespiratory systems (i.e. we did not examine embryos in this study), the reason for the slight increases in juvenile and subadult ventilation rates compared to adults, particularly in males, is unclear (Fig. 4). Given that there was only one juvenile female in the study, it is difficult to determine if there is physiological significance to these results. During these ventilation counts, we did observe that sharks were typically at rest for the first hour of the trials until ~30°C was reached. In many cases, from 30°C to the CT_max_ endpoint, sharks became more agitated and active, which is consistent with previous teleost work (Lutterschmidt and Hutchison, 1997; Wells *et al.,* 2016; Firth *et al.,* 2021). Overall, because we did not detect any differences in CT_max_ across life stages or between sexes, our data suggest that when a singular metric of upper thermal tolerance is used, upper thermal tolerance is conserved.

### Body size and CT_max_

Beyond specific life history stages and the associated energetic investments, body size itself also plays a role in upper thermal tolerance. Core body temperatures of large-bodied fishes heat more slowly than in smaller fishes due to lower surface area to volume ratios (Stevens and Fry, 1974; Zhang and Kieffer, 2014; Messmer *et al.,* 2017). Many studies have found small changes in CT_max_ with increasing body size, including both positive (Ziegeweid *et al.,* 2008; Zhang and Kieffer, 2014; Clark *et al.,* 2017) and negative relationships (e.g. Komoroske *et al.,* 2014; Clark *et al.,* 2017; Messmer *et al.,* 2017; Leiva *et al.,* 2019), and it is possible that differences in thermal ramping rates partially explain these differences. To fully clarify why the relationship between upper thermal tolerance, life history stage and body size is conflicting across the literature, future studies should include a full range of life stages, an appropriate thermal ramping rate, the same end point across stages and validate the reproductive status of adults.

### Implications and future directions

Attention to experimental design and implementation of CT_max_ estimates will be key to furthering our understanding of elasmobranch upper thermal tolerance. For example, in Gervais et al. (2018), Heron Island reef flat epaulette sharks demonstrated seasonal plasticity in CT_max_, where values were 2.93°C higher in the summer compared to the winter. Instead, our CT_max_ estimates averaged at 36.22°C (±0.36°C s.d.), which was 1.17°C degrees lower than expected for the spring season based on Gervais et al. (2018). However, in our study, we chose a slower thermal ramping rate for assays of 0.1°C min^−1^, whereas Gervais et al. (2018) used 0.26°C min^−1^. This methodological difference is likely why we found lower CT_max_ values than expected, because slower ramping rates typically produce lower CT_max_ end points (Messmer *et al.,* 2017; Kingsolver and Umbanhowar, 2018; Illing *et al.,* 2020). Therefore, selecting appropriate and consistent thermal ramping rates that allow the internal body temperature to mirror the thermal ramping assay is an important consideration moving forward with both inter- and intra-specific comparisons of CT_max_ among large-bodied fishes.

Considering which physiological metric to use when assessing upper thermal tolerance also warrants more investigation. For example, median lethal temperature (LT50) is also an excellent estimate of upper thermal tolerance in fishes (Pottier *et al.,* 2022), and would be easier to implement than CT_max_ in embryonic stages. However, given the lethal nature of this method, it may not be appropriate for studies on wild-caught elasmobranchs. Thermal effects on metabolic rate may also be a useful, non-lethal method to assess changes in organismal energetic costs. Adult epaulette shark metabolic costs do not differ between current day spring and summer mean water temperatures (25°C versus 28°C) (Wheeler *et al.,* 2022), but prolonged, chronic exposure to future ocean warming temperatures of 31°C does reduce physiological performance of embryos and neonates (Wheeler *et al.,* 2021). Indeed, chronic exposure to elevated temperatures that are well below CT_max_ may provide a better picture of sublethal effects of ocean warming across ontogeny.

Epaulette sharks are thought to reach maturity in 2–4 years (Vanderwright *et al.,* 2021), which for an elasmobranch, is a relatively short generation time. This may provide some scope for intergenerational adaptation in parallel to ocean warming within this species; however, most other elasmobranchs have longer generation times and may not keep pace, which could render species in this group particularly vulnerable as warming continues. Moving forward from this study, researchers can now refine experimental designs to better elucidate the thermal tolerance limits and overall physiological performance of this and other elasmobranch species in the context of continued ocean warming.

## Funding

This work was supported by a Save Our Seas Foundation small grant, JCU Postgraduate Research Scholarships (to C.R.W. and B.J.L.), the Australian Research Council (ARC) Centre of Excellence for Coral Reef Studies (to J.L.R.) and an anonymous donor to the New England Aquarium for funds that contributed equipment used in this study.

## Author Contributions

This project was conceived and designed by C.R.W., J.W.M. and J.L.R. Data were collected and analysed by C.R.W., B.J.L. and J.L.R. All authors were involved in the preparation of the manuscript.

## Data Availability Statement

The data underlying this article are available from Research Data JCU at James Cook University at https://doi.org/10.25903/zqvp-6h47.

## Supplementary Material

Web_Material_coac074

## References

[ref1] Andreassen AH , Hall P, Khatibzadeh P, Jutfelt F, Kermen F (2022) Brain dysfunction during warming is linked to oxygen limitation in larval zebrafish. Proc Natl Acad Sci U S A 119: e2207052119. 10.1073/pnas.2207052119. PMC9522358 36122217

[ref2] Angilletta Jr MJ (2009) Thermal Adaptations. Oxford University Press, New York, pp. 1–18, 10.1093/acprof:oso/9780198570875.001.1.

[ref3] Auer SK , Agreda E, Chen AH, Irshad M, Solowey J (2021) Late-stage pregnancy reduces upper thermal tolerance in a live-bearing fish. J Therm Biol 99: 103022. 10.1016/j.jtherbio.2021.103022. 34420649

[ref4] Awruch CA , Frusher SD, Stevens JD, Barnett A (2012) Movement patterns of the draughtboard shark *Cephaloscyllium laticeps* (Scyliorhinidae) determined by passive tracking and conventional tagging. J Fish Biol 80: 1417–1435. 10.1111/j.1095-8649.2012.03249.x. 22497391

[ref4a] Bates D , Mächler M, Bolker B, Walker S (2015) Fitting linear mixed-effects models using lme4. J Stat Softw 67:1–48. 10.18637/jss.v067.i01

[ref5] Bouyoucos IA , Morrison PR, Weideli OC, Jacquesson E, Planes S, Simpfendorfer CA, Brauner CJ, Rummer JL (2020) Thermal tolerance and hypoxia tolerance are associated in blacktip reef shark (*Carcharhinus melanopterus*) neonates. J Exp Biol 223: jeb221937. 10.1242/jeb.221937. 32694185

[ref6] Clark TD , Roche DG, Binning SA, Speers-Roesch B, Sundin J (2017) Maximum thermal limits of coral reef damselfishes are size dependent and resilient to near-future ocean acidification. J Exp Biol 220: 3519–3526. 10.1242/jeb.162529. 28754716

[ref7] Conrath CL , Musick JA (2012) Reproductive biology of elasmobranchs. In J Carrier, J Musick, eds, Biology of Sharks and Their Relatives, Ed2nd. CRC Press, Boca Raton, pp. 291–311.

[ref8] Dabruzzi TF , Bennett WA, Rummer JL, Fangue NA (2013) Juvenile ribbontail stingray, *Taeniura lymma* (Forsska 1775) (Chondrichthyes, Dasyatidae), demonstrate a unique suite of physiological adaptations to survive hyperthermic nursery conditions. Hydrobiologia 701: 37–49. 10.1007/s10750-012-1249-z.

[ref9] Dahlke FT , Wohlrab S, Butzin M, Pörtner H-O (2020) Thermal bottlenecks in the life cycle define climate vulnerability of fish. Science 369: 65–70. 10.1126/science.aaz3658. 32631888

[ref10] Dudgeon CL , Corrigan S, Yang L, Allen GR, Erdmann MV, Fahmi SHY, White WT, Naylor GJP (2019) Walking, swimming or hitching a ride? Phylogenetics and biogeography of the walking shark genus *Hemiscyllium*. Mar Freshw Res 71: 1107–1117. 10.1071/MF19163.

[ref11] Dulvy NK , Pacoureau N, Rigby CL, Pollom RA, Jabado RW, Ebert DA, Finucci B, Pollock CM, Cheok J, Derrick DH et al. (2021) Overfishing drives over one-third of all sharks and rays toward a global extinction crisis. Curr Biol 31: 4773–4787.e8 j.cub.2021.08.062. 10.1016/j.cub.2021.08.062. 34492229

[ref12] Fangue NA , Bennett WA (2003) Thermal tolerance responses of laboratory-acclimated and seasonally acclimatized Atlantic stingray, *Dasyatis sabina*. Copeia 2: 315–325.

[ref13] Firth BL , Drake DAR, Power M (2021) Seasonal and environmental effects on upper thermal limits of eastern sand darter *(Ammocrypta pellucida)*. Conserv Physiol 9: coab057. 10.1093/conphys/coab057. PMC8336138 35928053

[ref14] Fox-Kemper B , Hewitt HT, Xiao C, Adalgeirsdottir G, Drijfhout SS, Edwards TL, Golledge NR, Hemer M, Kopp RE, Krinner G et al. (2021) Ocean, cryosphere and sea level change. In: Masson-Delmotte V, Zhai P, Pirani A, Connors SL, Pean C, Berger S, Caud N, Chen Y, Goldfarb L, Gomis MI et al., eds. Climate Change 2021: The Physical Science Basis. Contribution of Working Group I to the Sixth Assessment Report of the Intergovernmental Panel on Climate Change. Cambridge University Press, Cambridge, United Kingdom and New York, NY, USA, pp. 1211–1362, 10.1017/9781009157896.011.

[ref15] Freitas V , Cardoso JFMF, Lika K, Peck MA, Campos J, Kooijman SALM, van der Veer HW (2010) Temperature tolerance and energetics: a dynamic energy budget-based comparison of North Atlantic marine species. Phil Trans R Soc B 365: 3553–3565. 10.1098/rstb.2010.0049. 20921053 PMC2981968

[ref16] Gendron SM , Menzies S (2004) Elasmobranch acclimation and introduction. In M Smith, D Warmolts, D Thoney, R Hueter, eds, Elasmobranch Husbandry Manual: Captive Care of Sharks, Rays, and Their Relatives. Ohio Biological Survey, Inc, Columbus, pp. 151–162.

[ref17] Gervais CR , Nay TJ, Renshaw G, Johansen JL, Steffensen JF, Rummer JL (2018) Too hot to handle? Using movement to alleviate effects of elevated temperatures in a benthic elasmobranch, Hemiscyllium ocellatum. Mar Biol 165: 162. 10.1007/s00227-018-3427-7.

[ref18] Gunderson AR , Stillman JH (2015) Plasticity in thermal tolerance has limited potential to buffer ectotherms from global warming. Proc R Soc B 282: 20150401. 10.1098/rspb.2015.0401. PMC4455808 25994676

[ref19] Hayward A , Gillooly JF (2011) The cost of sex: quantifying energetic investment in gamete production by males and females. PLoS One 6: e16557. 10.1371/journal.pone.0016557. PMC3026017 21283632

[ref20] Heupel MR , Whittier JM, Bennett MB (1999) Plasma steroid hormone profiles and reproductive biology of the epaulette shark, *Hemiscyllium ocellatum*. J Exp Zool 284: 586–594. 10.1002/(SICI)1097-010X(19991001)284:5<586::AID-JEZ14>3.0.CO;2-B. 10469996

[ref21] Illing B , Downie AT, Beghin M, Rummer JL (2020) Critical thermal maxima of early life stages of three tropical fishes: effects of rearing temperature and experimental heating rate. J Therm Biol 90: 102582. 10.1016/j.jtherbio.2020.102582. 32479385

[ref22] Johnson CR (1976) Diel variation in the thermal tolerance of *Gambusia affinis affinis* (Pisces: Poeciliidae). Comp Biochem Physiol A 55: 337–340. 10.1016/0300-9629(76)90056-6. 9246

[ref23] Kingsolver JG , Umbanhowar J (2018) The analysis and interpretation of critical temperatures. J Exp Biol 221: jeb167858. 10.1242/jeb.167858. 29724777

[ref24] Kneebone J , Sulikowski J, Knotek R, McElroy WD, Gervelis B, Curtis T, Jurek J, Mandelman J (2020) Using conventional and pop-up satellite transmitting tags to assess the horizontal movements and habitat use of thorny skate (*Amblyraja radiata*) in the Gulf of Maine. ICES J Mar Sci 77: 2790–2803. 10.1093/icesjms/fsaa149.

[ref25] Komoroske LM , Connon RE, Lindberg J, Cheng BS, Castillo G, Hasenbein M, Fangue NA (2014) Ontogeny influences sensitivity to climate change stressors in an endangered fish. Conserv Physiol 2: cou008. 10.1093/conphys/cou008. PMC4806739 27293629

[ref26] Komsta L (2022) Outliers: tests for outliers. R package version 0.15. https://CRAN.R-project.org/package=outliers.

[ref27] Leiva FP , Calosi P, Verberk WCEP (2019) Scaling of thermal tolerance with body mass and genome size in ectotherms: a comparison between water- and air-breathers. Phil Trans R Soc B 374: 20190035. 10.1098/rstb.2019.0035. 31203753 PMC6606457

[ref28] Lenth RV (2022) Emmeans: estimated marginal means, aka least-squares means. R package version 1.7.2. https://CRAN.R-project.org/package=emmeans.

[ref29] Lutterschmidt WI , Hutchison VH (1997) The critical thermal maximum: history and critique. Can J Zool 75: 1561–1574. 10.1139/z97-783.

[ref30] Messmer V , Pratchett MS, Hoey AS, Tobin AJ, Coker DJ, Cooke SJ, Clark TD (2017) Global warming may disproportionately affect larger adults in a predatory coral reef fish. Glob Change Biol 23:2230–2240. 10.1111/gcb.13552. 27809393

[ref31] Morgan R , Finnøen MH, Jutfelt F (2018) CT_max_ is repeatable and doesn’t reduce growth in zebrafish. Sci Rep 8: 7099. 10.1038/s41598-018-25593-4. 29740113 PMC5940882

[ref32] Morgan R , Sundin J, Finnøen MH, Dresler G, Vendrell MM, Dey A, Sarkar K, Jutfelt F (2019) Are model organisms representative for climate change research? Testing thermal tolerance in wild and laboratory zebrafish populations. Conserv Physiol 7: coz036. 10.1093/conphys/coz036. 31249690 PMC6589993

[ref33] Nay TJ , Longbottom RJ, Gervais CR, Johansen JL, Steffensen JF, Rummer JL, Hoey AS (2021) Regulate or tolerate: thermal strategy of a coral reef flat resident, the epaulette shark, *Hemiscyllium ocellatum*. J Fish Biol 98: 723–732. 10.1111/jfb.14616. 33206373

[ref34] Ospina AF , Mora C (2004) Effect of body size on reef fish tolerance to extreme low and high. Environ Biol Fishes 70: 339–343. 10.1023/B:EBFI.0000035429.39129.34.

[ref35] Penney CM , Burness G, Tabh JKR, Wilson CC (2021) Limited transgenerational effects of environmental temperatures on thermal performance of a cold-adapted salmonid. Conserv Physiol 9: coab021. 10.1093/conphys/coab021. PMC8071478 33959288

[ref36] Pereira Santos C , Sampaio E, Pereira BP, Pegado MR, Borges FO, Wheeler CR, Bouyoucos IA, Rummer JL, Santos CF, Rosa R (2021) Elasmobranch responses to experimental warming, acidification, and oxygen loss—a meta-analysis. Front Mar Sci 8: 735377. 10.3389/fmars.2021.735377.

[ref38] Pörtner H-O , Farrell AP (2008) Physiology and climate change. Science 322: 690–692. 10.1126/science.1163156. 18974339

[ref39] Pörtner H-O , Peck MA (2010) Climate change effects on fishes and fisheries: towards a cause-and-effect understanding. J Fish Biol 77: 1745–1779. 10.1111/j.1095-8649.2010.02783.x. 21078088

[ref40] Pottier P , Burke S, Drobniak SM, Nakagawa S (2022) Methodological inconsistencies define thermal bottlenecks in fish life cycle: a comment on Dahlke *et al.,* 2020. Evol Ecol 36: 287–292. 10.1007/s10682-022-10157-w.

[ref41] Przeslawski R , Byrne M, Mellin C (2015) A review and meta-analysis of the effects of multiple abiotic stressors on marine embryos and larvae. Glob Chang Biol 21: 2122–2140. 10.1111/gcb.12833. 25488061

[ref42] R Core Team (2021) R: A language and environment for statistical computing. R Foundation for Statistical Computing, Vienna, https://www.R-project.org/.

[ref43] Recsetar MS , Zeigler MP, Ward DL, Bonar SA, Caldwell CA (2012) Relationship between fish size and upper thermal tolerance. T Am Fish Soc 141: 1433–1438. 10.1080/00028487.2012.694830.

[ref44] Seebacher F , White CR, Franklin CE (2015) Physiological plasticity increases resilience of ectothermic animals to climate change. Nat Clim Chang 5: 61–66. 10.1038/nclimate2457.

[ref45] Stevens ED , Fry FEJ (1974) Heat transfer and body temperatures in non-thermoregulatory teleosts. Can J Zool 52: 1137–1143. 10.1139/z74-152. 4417360

[ref46] VanderWright WJ , Dudgeon CL, Erdmann MV, Sianipar A, Dulvy NK (2022) Extinction risk and the small population paradigm in the micro-endemic radiation of epaulette sharks. In: DellaSala DA, Goldstein, MI, eds. Imperiled: The Encyclopedia of Conservation. Elsevier, Amsterdam, Netherlands pp. 752–762. 10.1016/b978-0-12-821139-7.00130-6.

[ref47] Vilmar M , Di Santo V (2022) Swimming performance of sharks and rays under climate change. Rev Fish Biol Fisheries 32: 765–781. 10.1007/s11160-022-09706-x.

[ref48] Wells ZRR , McDonnell LH, Chapman LJ, Fraser DJ (2016) Limited variability in upper thermal tolerance among pure and hybrid populations of a cold-water fish. Conserv Physiol 4: cow063. 10.1093/conphys/cow063. PMC5156897 27990291

[ref49] Wheeler CR , Kneebone J, Heinrich D, Strugnell JM, Mandelman JW, Rummer JL (2022) Diel rhythm and thermal independence of metabolic rate in a benthic shark. J Biol Rhythms 37: 484–497. 10.1177/07487304221107843. 35822624

[ref50] Wheeler CR , Rummer JL, Bailey B, Lockwood J, Vance S, Mandelman JW (2021) Future thermal regimes for epaulette sharks (*Hemiscyllium ocellatum*): growth and metabolic performance cease to be optimal. Sci Rep 11: 454. 10.1038/s41598-020-79953-0. 33436769 PMC7804200

[ref51] Wood SN (2011) Fast stable restricted maximum likelihood and marginal likelihood estimation of semiparametric generalized linear models. J R Stat Soc B 73: 3–36. 10.1111/j.1467-9868.2010.00749.x.

[ref52] Zhang Y , Kieffer JD (2014) Critical thermal maximum (CT_max_) and hematology of shortnose sturgeons *(Acipenser brevirostrum)* acclimated to three temperatures. Can J Zool 92: 215–221. 10.1139/cjz-2013-0223.

[ref53] Ziegeweid JR , Jennings CA, Peterson DL (2008) Thermal maxima for juvenile shortnose sturgeon acclimated to different temperatures. Environ Biol Fishes 82: 299–307. 10.1007/s10641-007-9292-8.

