# Supplementary material for: The upper thermal limit of epaulette sharks (Hemiscyllium ocellatum) is conserved across three life history stages, sex and body size
Source: Conserv Physiol. 2022 Dec 28;10(1):coac074. doi: 10.1093/conphys/coac074 (PMC9795165; doi:10.1093/conphys/coac074)
Supplement: Web_Material_coac074 [file web_material_coac074.docx]

**Title:** The upper thermal limit of epaulette sharks (*Hemiscyllium ocellatum*) is conserved across three life history stages, sex, and body size

**Authors:** Carolyn R. Wheeler^1,2^ (ORCID: /0000-0001-9976-8420);

Bethan J. Lang^1^;

John W. Mandelman ^2,3^;

Jodie L. Rummer^1,4^ (ORCID: /0000-0001-6067-5892)

^1^ARC Centre of Excellence for Coral Reef Studies, James Cook University, Townsville, Queensland 4814, Australia

^2^School for the Environment, The University of Massachusetts Boston, Boston, Massachusetts 02125, USA

^3^Anderson Cabot Center for Ocean Life, New England Aquarium, Boston, Massachusetts 02110, USA

^4^College of Science and Engineering, James Cook University, Townsville, Queensland 4814, Australia

**Corresponding author:** Carolyn Wheeler: carolyn.wheeler23@gmail.com; + 61 0480 129 737; 1 James Cook Drive Douglas, QLD 4814 AUS

**Keywords:** Chondrichthyes, critical thermal maximum, epaulette shark, thermal tolerance, ocean warming

**S0. Test for outliers**

1. Outlier in CT_max_:


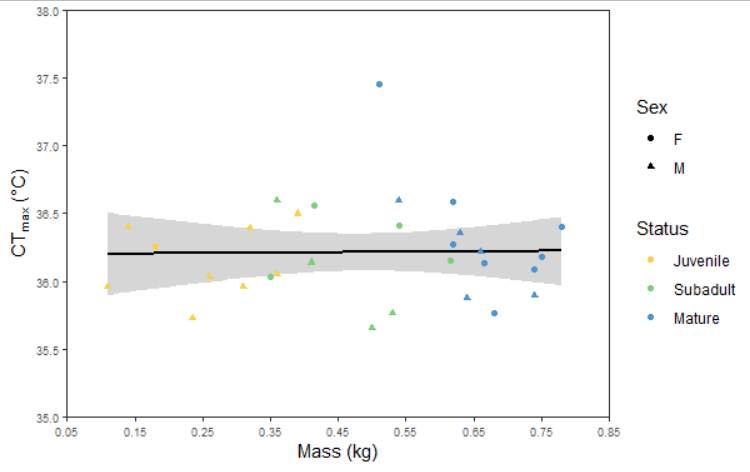


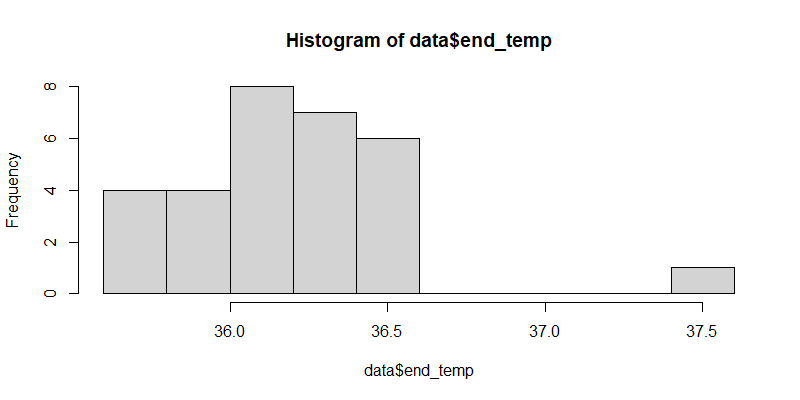


1. Dixon’s Q test:

Q = 0.50595, p-value = 0.001172

*Accept the alternative hypothesis: highest value 37.45 is an outlier.*

1. Outlier removed:

- Individual HO-325, a mature female was removed.


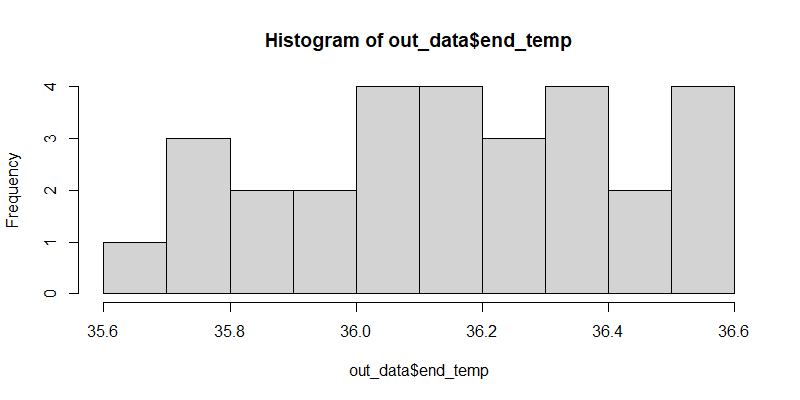


**S1. Experimental parameter effects on CT_max_**

lm(CT_max_ ~ number of holding days + trial starting temperature*time of day of trial)

| **Term** | **Sum of squares** | **df** | **F statistic** | **p-value** |
| --- | --- | --- | --- | --- |
| Holding days | 0.02635 | 1 | 0.328933 | 0.571624 |
| Trial starting temp | 0.040435 | 1 | 0.504751 | 0.484267 |
| Time of day of trial | 0.014232 | 1 | 0.177664 | 0.67714 |
| Start temp*time of day | 0.113742 | 1 | 1.419858 | 0.245077 |
| Residuals | 1.922588 | 24 | NA | NA |

**S2. Life history stage, sex, and body size effects on CT_max_**

lm(centred CT_max_ ~ status + sex + mass + life history stage*sex*mass)

| **Term** | **Sum of squares** | **df** | **F statistic** | **p-value** |
| --- | --- | --- | --- | --- |
| Life history stage | 0.081812 | 2 | 0.601321 | 0.558732 |
| Mass | 0.090298 | 1 | 1.327384 | 0.264344 |
| Sex | 0.190384 | 1 | 2.798658 | 0.111634 |
| Life history stage*mass | 0.6207 | 2 | 4.562174 | 0.024959 |
| Life history stage*sex | 0.425097 | 2 | 3.124483 | 0.068425 |
| Mass*sex | 0.410419 | 1 | 6.033195 | 0.024426 |
| Life history stage*mass*sex | 0.033445 | 1 | 0.491641 | 0.492162 |
| Residuals | 1.224482 | 18 | NA | NA |

**S3. CT_max_ trial activity proportions**

glmer(activity type ~ status + sex + (1|ID), family= binomial(link= “logit”))

| **Random effect** | **Variance** | **Std. Dev.** |  |  |
| --- | --- | --- | --- | --- |
| ID | 0.6632 | 0.8143 |  |  |
| **Fixed effects** | **Estimate** | **Std. Error** | **Z value** | **Pr(>\|z\|)** |
| Intercept | -2.7838 | 0.6405 | -4.346 | 1.39e-05 |
| Status:Mature | 0.6646 | 0.5937 | 1.120 | 0.2629 |
| Status:Subadult | 1.1167 | 0.6307 | 1.771 | 0.0766 |
| Sex:Male | 0.3642 | 0.4773 | 0.763 | 0.4454 |

Type II Wald Chi squared tests

| **Term** | **Chisq.** | **df** | **Pr(>Chisq)** |
| --- | --- | --- | --- |
| Status | 3.1385 | 2 | 0.2082 |
| Sex | 0.5823 | 1 | 0.4454 |

**S4. Resting ventilation rate generalized additive model**

gam(ventilation rate ~ status + sex + status:sex + s(exp_time, k= -1, by= status:sex), correlation= corAR1())

| **Term** | **Estimate** | **Std. Error** | **t value** | **Pr(>\|t\|)** |
| --- | --- | --- | --- | --- |
| Intercept | 38.861 | 1.713 | 22.680 | <2e-16 |
| Status:subadult | 8.120 | 2.070 | 3.922 | 0.000107 |
| Status:mature | 3.622 | 1.833 | 1.976 | 0.049045 |
| Sex:male | 3.641 | 1.852 | 1.966 | 0.050154 |
| Status:subadult *Sex:male | -8.424 | 2.377 | -3.544 | 0.000453 |
| Status:mature*Sex:male | -8.093 | 2.143 | -3.777 | 0.000189 |

Type II Wald Chi squared tests

| **Term** | **F** | **df** | **Pr(>Chisq)** |
| --- | --- | --- | --- |
| Status | 9.091 | 2 | 0.000145 |
| Sex | 3.866 | 1 | 0.050154 |
| Status:Sex | 8.049 | 2 | 0.000389 |

Estimated marginal means comparisons: ~Status + (1|Sex)

| **Sex** | **contrast** | **estimate** | **std.error** | **df** | **statistic** | **adj.p.value** |
| --- | --- | --- | --- | --- | --- | --- |
| Female | Juvenile - Subadult | -14.697 | 4.266606 | 318.8413 | -3.44465 | 0.001871 |
|  | Juvenile - Mature | -4.83555 | 2.057458 | 318.8413 | -2.35025 | 0.050559 |
|  | Subadult - Mature | 9.8614 | 4.070024 | 318.8413 | 2.422934 | 0.042075 |
| Male | Juvenile - Subadult | 0.232448 | 1.687651 | 318.8413 | 0.137735 | 0.989596 |
|  | Juvenile - Mature | 5.054203 | 1.326929 | 318.8413 | 3.808946 | 4.90E-04 |
|  | Subadult - Mature | 4.821754 | 1.601639 | 318.8413 | 3.010512 | 0.007908 |
